# FATS is a transcriptional target of p53 and associated with antitumor activity

**DOI:** 10.1186/1476-4598-9-244

**Published:** 2010-09-16

**Authors:** Xifeng Zhang, Qian Zhang, Jun Zhang, Li Qiu, Shuang-shuang Yan, Juling Feng, Yan Sun, Xingxu Huang, Karen H Lu, Zheng Li

**Affiliations:** 1 Department of Biochemistry and Molecular Biology, Key Laboratory of Ministry of Education for Breast Cancer Prevention and Treatment, Tianjin Medical University Cancer Institute and Hospital, Tianjin 300060, China; 2 Department of Gynecologic Oncology, University of Texas MD Anderson Cancer Center, Houston, Texas 77030, USA; 3 Jinan University Medical School, Guangzhou 510632, China; 4 Department of Pathology, Tianjin Medical University Cancer Institute and Hospital, Tianjin 300060, China; 5 Model Animal Research Center, Nanjing University, Nanjing 210093, China

## Abstract

Frequent mutations of p53 in human cancers exemplify its crucial role as a tumor suppressor transcription factor, and p21, a transcriptional target of p53, plays a central role in surveillance of cell-cycle checkpoints. Our previous study has shown that FATS stabilize p21 to preserve genome integrity. In this study we identified a novel transcript variant of FATS (GenBank: GQ499374) through screening a cDNA library from mouse testis, which uncovered the promoter region of mouse FATS. Mouse FATS was highly expressed in testis. The p53-responsive elements existed in proximal region of both mouse and human FATS promoters. Functional study indicated that the transcription of FATS gene was activated by p53, whereas such effect was abolished by site-directed mutagenesis in the p53-RE of FATS promoter. Furthermore, the expression of FATS increased upon DNA damage in a p53-dependent manner. FATS expression was silent or downregulated in human cancers, and overexpression of FATS suppressed tumorigenicity *in vivo *independently of p53. Our results reveal FATS as a p53-regulated gene to monitor genomic stability.

## Findings

Malfunction of the p53 pathway is an almost universal hallmark of human tumors, and mutations selected for tumorigenesis are usually single base substitutions that result in amino acid substitutions in the DNA-binding domain of p53 [1,2]. As a transcription factor, p53 controls multiple cellular functions by inducing or repressing target genes with p53 response elements (REs). Among main outcomes after the activation of p53, cell-cycle arrest permits repair processes to act and plays a key role in surveillance of genome stability, which is executed predominately by a downstream target gene p21 [3,4].

Fragile-site associated tumor suppressor (FATS) is a newly identified candidate tumor suppressor whose gene locus is frequently deleted in spontaneous tumors. Strikingly, the deletion frequency of FATS gene in tumors increases further after treatment of low dose ionizing radiation (IR), which is tightly linked to DNA-damage-induced tumorigenesis and loss of FATS-mediated inhibition of p21 turnover [5]. However, it's unknown whether crosstalk occurs between p53-p21 pathway and FATS-p21 pathway.

To understand better the functional network of FATS, we investigated the transcriptional regulation of FATS. In this study, we identified a novel FATS transcript variant with a distinct 5' untranslated region (UTR). The mRNA level of mouse FATS was highest in testis. Furthermore, we found that the transcription of FATS was activated by p53, and that FATS possessed anticancer activity independently of p53.

**Figure 1:**
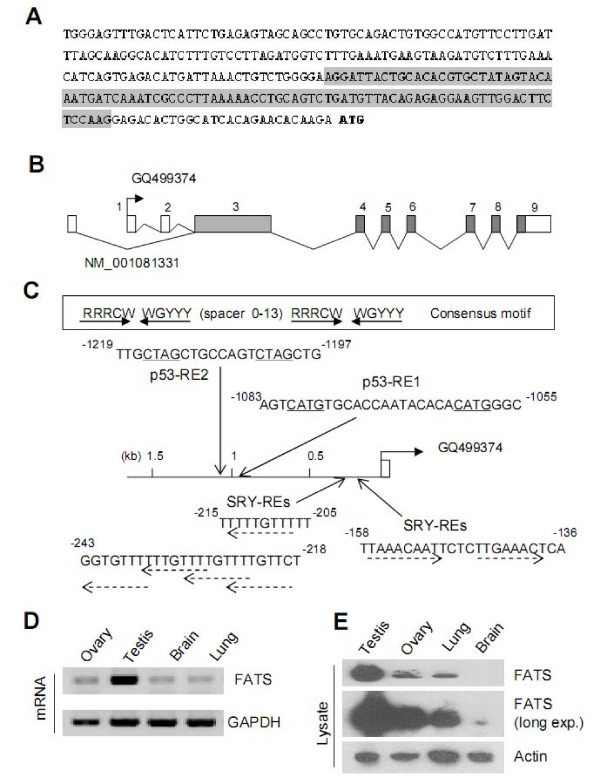
**Identification of a FATS transcription variant with high expression in mouse testis**. (A) The sequence of 5'-UTR in FATS mRNA (GenBank accession number: GQ499374) through screening a mouse testis plasmid cDNA library (Stratagene #975304). In brief, the non-coding sequence ahead of the first coding exon of FATS gene was amplified by PCR using the primers specific to pMyr XR vector (Stratagene) and FATS coding region, respectively, and subjected to DNA sequencing subsequently. Primer sequences were as follows: pMyr-fw, 5'-ATGGGGAGTAGCAAGAGCAA-3'; FATS-rv, 5'-TCTTTTCATCAATCAGCCGG-3'. Similar approach was applied to verify the mRNA sequence downstream of the first coding exon. Primer sequences were as follows: FATS-fw, 5'-CATATTCCCGGCTGGAGTTA-3'; pMyr-rv, 5'-CTTTTCGGTTAGAGCGGATG-3'. The sequence of exon 2 is shaded, and the start codon ATG is in bold letters. (B) The genomic organization of FATS gene. FATS transcript GQ499374 differed from a previously submitted transcription variant (GenBank accession number: NM_001081331) only in 5' UTR. The coding regions of exons were shaded. (C) The p53-responsive elements (p53-RE) in mouse FATS promoter. The p53-binding site is composed of a half-site RRRCWWGYYY followed by a spacer, usually composed of 0-13 base pairs, which is then followed by a second half-site RRRCWWGYYY sequence [3,4]. R, purine; Y, pyrimidine; W, adenine or thymine. The responsive elements of the testis-determining factor SRY (SRY-REs) in mouse FATS promoter were analyzed by TFsearch program http://www.cbrc.jp/research/db/TFSEARCH.html. (D) The expression level of FATS mRNA in mouse testis and ovary was determined by RT-PCR. (E) FATS protein levels in mouse tissues. Exp.: exposure.

The NH2-terminal region of FATS, encoded by the largest coding exon that is susceptible to DNA damage and frequently deleted in tumors, plays a critical role in FATS-mediated regulation of p21 [5]. In an effort to identify the full-length mRNA of FATS, we took advantage of a mouse testis plasmid cDNA library based on the sequence of the first coding exon and cloning vector. Rapid amplification of cDNA Ends (RACE) was then applied to obtain the full-length sequence of FATS mRNA. The results of library screening revealed a previously unknown transcript variant that possessed a novel 5' untranslated region (UTR), which was spliced from two exons (Figure 1A and 1B). This transcript variant of FATS (GenBank accession number: GQ499374) shared all the coding exons with a previously existing transcript in GenBank (NM_001081331), whose exon 1 was not present in the cDNA clone identified or undetectable by RT-PCR in our study (Figure 1B and data not shown). Identification of FATS transcript variant GQ499374 revealed a new promoter region of FATS gene.

Given that p53 insufficiency, which also impairs DNA repair, markedly increases the risk of FATS deletion and insufficiency [3,5,6], we evaluated whether FATS might be a transcription target of p53. Bioinformatics analysis indicated the presence of two p53-REs within 1.3 kb upstream of transcriptional start site (TSS) of mouse FATS (GQ499374), but not within 2.0 kb upstream of TSS of transcript variant NM_001081331 (Figure 1C and data not shown). Interestingly, FATS promoter also contains multiple AACAAT consensus motifs (Figure 1C), which are binding sites of a family of testis-determining factor SRY [7,8]. Consistently, the expression level of FATS mRNA was much higher in mouse testis in comparison to that in ovary, brain and lung (Figure 1D). To validate the high expression of FATS in testis, lysates from these tissues were subjected to immunoblotting using an antibody specific to FATS. Indeed, the expression level of FATS protein was the highest in testis (Figure 1E). Moreover, the expression levels of FATS protein in ovary, lung and brain were not proportional to their mRNA levels (Figure 1D and 1E), suggesting the involvement of tissue-specific and posttranscriptional regulation of FATS expression.

**Figure 2:**
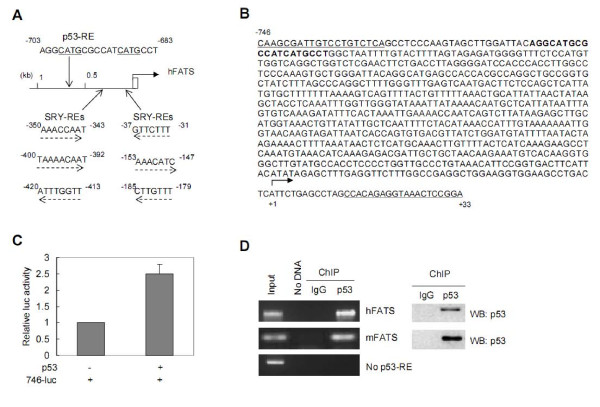
**FATS is a transcriptional target of p53**. (A) The p53-RE sequence in the promoter of human FATS gene (GenBank accession number: NM_001004298). The sequences of SRY-REs were also shown. (B) The sequence (-746 to +33) around TSS of human FATS was amplified by PCR and a luciferase reporter 746-luc was constructed subsequently. The sequence of a p53-RE was highlighted, and the primer sequences were underlined. (C) 746-luc (0.5 μg) was transfected with or without p53 (0.1 μg) by Lipofactamine (Invitrogen) into U87 cells carrying wild-type p53. A reporter pRL-TK (0.1 μg, Promega) was used as internal control for trasnfection. After 24 h, dual luciferase assay was performed [10], and the firefly lucifease activity derived from 746-luc was normalized by *Renilla *luciferase activity derived from pRL-TK. Results of three independent experiments were averaged and plotted as relative luciferase activity. Error bars refer to standard deviation (± SD). (D) U87 or MEF cells were treated with 1% formaldehyde for 10 min, respectively, and subjected to chromatin immunoprecipitation (ChIP) analysis. Primer sequences were 5'-CAAGCGATTGTCCTGTCTCA-3' and 5'-GAGACCAGCCTGACCAACAT-3' (human FATS); 5'-CCGCCTCAAAAGGATAAGGT-3' and 5'-GCAGGAACAATTACAACAATATGC-3' (mouse FATS), 5'-GTGTCCACCTCAGGGTGTCT-3' and 5'-CTTCAGGGCTTCGCTTATTG-3' (no p53-RE at hFATS exon 3).

There is only one p53-RE (at -703 to -683 bp) within the promoter region of human FATS, whose promoter also contains multiple SRY-REs (Figure 2A). To determine whether this p53-RE might be functional, we cloned human FATS promoter and generated a luciferase reporter 746-luc (Figure 2B). The reporter 746-luc was transfected alone or co-transfected with a p53-expressing vector into U87 cells (p53 wild-type) derived from human glioblastoma. The luciferase activity driven by FATS promoter was significantly higher in the presence of p53 (Figure 2C), indicating that p53 can regulate FATS gene. To investigate whether p53 directly regulates the transcription of FATS, chromatin immunoprecipitation (ChIP) was performed to detect the physical binding of p53 to FATS promoter *in vivo*. Chromatins from human U87 cells or primary mouse embryonic fibroblast (MEF) cells were incubated with p53 antibody or IgG control, respectively. Purified DNA was then analyzed by PCR using primers specific for FATS promoter. PCR product was observed after immunoprecipitation with anti-p53 only from precipitated DNA containing p53-RE but not in the IgG ChIP or the "no DNA" PCR control (Figure 2D), indicating direct regulation of FATS transcription by p53.

**Figure 3:**
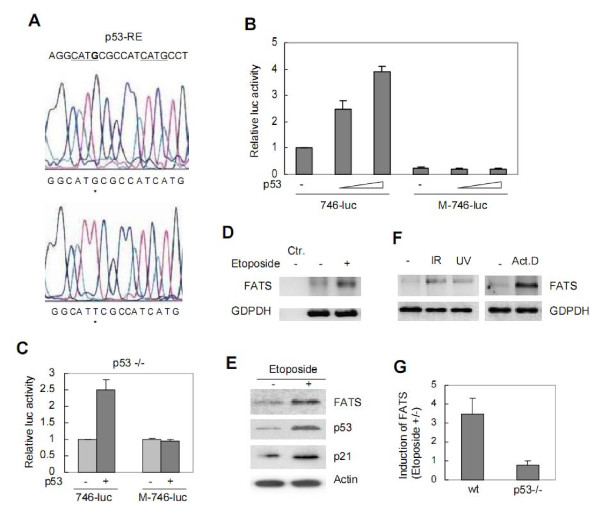
**Effect of site-specific mutagenesis and DNA damage on p53-mediated transactivation of FATS**. (A) Site-directed mutagenesis in p53-RE consensus to generate M-746-luc. (B) The reporters was tranfected alone, or co-transfected with increased amount of p53 (0.1 μg, 0.3 μg, respectively) into U87 cells. Dual luciferase activities were measured after 24 h. (C) Luciferase reporter assay in p53-null cells. (D) MEF cells were untreated or treated with etoposide (0.1 μg/ml) for 4 h. The total RNA was prepared and the expression of FATS mRNA was subsequently detected by RT-PCR. A negative control (Ctr.) with no reverse transcriptase was also showed. (E) MEF cells were untreated or treated with etoposide (0.1 μg/ml) for 6 h. Cell lysates were prepared and subjected to immunoblotting. (F) U87 cells were untreated or treated with ionizing radiation (IR, 10Gy), UV (4 kJ/m^2^), or actinomycin D (Act.D, 20 nM), respectively. The expression of FATS mRNA was detected by RT-PCR. (G) Induction of FATS protein in p53-null MEF cells with or without etoposide (0.1 μg/ml) treatment for 4 h.

In consensus motif of p53-RE, the most important bases for interactions with the p53 protein are the central CWWG nucleotides within each half-site, especially the C and G [3,9]. To gain further insight into the regulatory specificity of p53-mediated transactivation of FATS, we generated a reporter M-746-luc in which the G at the first central CWWG of p53-RE was changed to T through site-specific mutagenesis (Figure 3A). 746-luc or M-746-luc was transfected alone or co-transfected with p53 into U87 cells, and cell lysates were subjected to luciferase assay subsequently [10]. The luciferase activity driven by FATS promoter increased in the presence of p53 in a dose-dependent manner. However, the basal luciferase level after M-746-luc transfection alone in U87 cells was lower than that after 746-luc transfection, and site-directed mutagenesis in p53-RE abolished p53-mediated enhancement of luciferase activity derived from M-746-luc under the same conditions (Figure 3B). Consistently, the luciferase activity derived from 746-luc was not significantly higher than that from M-746-luc in p53-null cells. However, the luciferase activity driven by mutant, but not wild-type, FATS promoter, failed to increase after p53 transfection into p53-null cells (Figure 3C). These results demonstrated that p53 specifically regulated the transcription of FATS.

To provide a physiological context for our findings, we next examined whether the transcription of FATS might be stimulated after exposure to DNA-damaging agents, resulting from the activation of p53 [11]. MEF cells were either untreated or treated with etoposide, an inhibitor of DNA topoisomerase II. After 4 h, total RNA was prepared and subjected to reverse transcrtiption and polymerase chain reaction (RT-PCR) subsequently. Indeed, the mRNA level of FATS was significantly increased after etoposide treatment (Figure 3D), further supporting that FATS is a transcription target of p53 involved in DNA damage response. The induction of p53 protein after treatment of etoposide not only led to p21 induction but also accompanied the induction of FATS protein (Figure 3E). Furthermore, the FATS mRNA level increased after the treatment of other DNA damage agents such as ionizing radiation, UV and actinomycin D (Figure 3F). The induction of FATS after exposure to DNA-damaging agents was significantly compromised in p53-null cells (Figure 3G). Taken together, these results demonstrated that FATS was induced in the DNA damage response in a p53-dependent manner.

**Figure 4:**
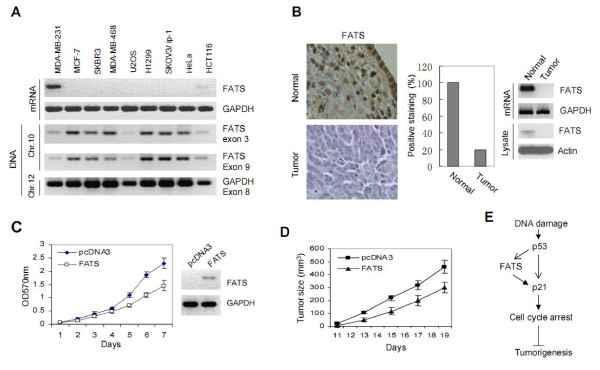
**The expression of FATS in human cancers and FATS-mediated tumor-suppressing activity**. (A) The mRNA levels of FATS in human cancer cell lines. The primers for FATS expression: 5'-CAATAAGCGAAGCCCTGAAG-3' and 5'-TTGGAGAGCTATCCCCAATG -3'. The same amount of genomic DNA (0.05 μg) was used for PCR amplification (25 cycles) simulteneously. Primer sequences were as follows: 5'-GTGTCCACCTCAGGGTGTCT-3' and 5'-CTTCAGGGCTTCGCTTATTG-3' (FATS exon 3); 5'-CCCATCAGAGAGGCCTGATA-3' (FATS exon 9); 5'-ACCCAGAAGACTGTGGATGG-3' and 5'-TTCTAGACGGCAGGTCAGGT-3' (GAPDH exon 8). Chr., Chromosome. (B) Immunohistochemistry of FATS in ovarian cancer samples. Representative slide photography showing intense staining of normal ovarian section whereas negative staining in 80% (n = 15) tested ovarian cancer samples. The percentage of positive staining of FATS was showed in right panel. Normal, N; Tumor, T. The expression of FATS in normal ovarian tissues and ovarian tumor samples were also examined by RT-PCR and immunoblotting. The representative images were shown in the right panel. (C) The effect of FATS on the growth of HeLa cancer cells *in vitro*. HeLa cells were stably transfected with FATS and an empty vector pcDNA3 (Invitrogen), respectively. The growth rates of HeLa-FATS and HeLa-pcDNA3 cells were measured by MTT assay [5]. The expression of FATS in HeLa-FATS cells was shown in the right panel. (D) *In vivo *tumorigenicity analysis of HeLa-FATS and HeLa-pcDNA3 cells. Xenograft tumor volumes in SCID mice were measured (n = 10 mice per group) using the formula: Volume = S × S × L/2, where S is the short length of the tumor in mm and L is the long length of the tumor in mm. (E) Schematic representation showing that p53-mediated transactivation of FATS maximizes the effect on maintaining p21 abundance, leading to the suppression of tumorigenesis.

We further evaluated the association of FATS with human cancers. The expression levels of FATS mRNA in nine cancer cell lines, including MDA-MB-231, SKBR3, MCF-7, MDA-MB-468 (breast cancer), U2OS (osteosarcoma), H1299 (lung cancer), SKOV3/ip-1 (ovarian cancer), HeLa (cervical cancer) and HCT116 (colon cancer) were detected by RT-PCR. FATS mRNA was undetectable in 7 out of 9 tested cell lines, and trace amount of FATS mRNA was expressed in HCT116 cells (Figure 4A). Two exons of FATS on chromosome 10 and one exon of GAPDH on chromosome 12 were simulteneously amplified by PCR using the same amount of genomic DNA. Homologous deletion of FATS gene in these cancer cells did not occur. However, the quantity of amplified FATS DNA fragment was significantly less in comparison with that of GAPDH DNA control in all these cancer cells (Figure 4A), indicating that the loss of mRNA expression of FATS is not only due to genomic deletion of coding strand in cancer genome, which is supported by loss of heterozygosity (LOH) studies [12,13], but also due to inhibition of gene transcription. Consistently, the expression of FATS protein was negative in 80% (n = 15) ovarian tumors, which was examined by immunohistochemistry, RT-PCR and immunoblotting (Figure 4B). These results, in agreement with frequent deletion of FATS locus in mouse tumors [5], further suggest the role of FATS as a tumor suppressor. To validate the function of FATS in suppression of tumorigenesis, we established a FATS-expressing stable cell line (HeLa-FATS) and its control (HeLa-pcDNA3) to examine the anti-tumor activities of FATS. The overexpression of FATS not only suppressed the growth of HeLa cancer cells *in vitro *(Figure 4C), but also suppressed the tumor growth in xenograft tumor models (Figure 4D). Because p53 is inactivated in HeLa cells, these results demonstrate that FATS is capable of suppressing tumor growth in the absence of functional p53.

The main outcomes after the activation of p53 include apoptosis, senescence or cell-cycle arrest. The first two are terminal for the cell, whereas cell-cycle arrest permits DNA damage to be repaired and genome integrity to be maintained in a stressed cell. Among hundreds of identified p53 targeting genes [3], p21 is a well-established transcriptional target of p53 to mediate the effect of cell-cycle arrest. Additionally, p21 is a major determinant of tumor suppression by p53, especially in case p53 loses its capacity in inducing apoptosis [14,15]. Our previous study reveals FATS as a novel regulator of p21, which promotes the protein stability of p21 to monitor cell-cycle checkpoints after DNA damage [5]. In this study, we showed that FATS, possessing anticancer activities by itself, was transactivated by p53. These findings indicate the crosstalk between FATS-mediated p21 stabilization and p53-mediated p21 transactivation, which control p21 abundance tighter (Figure 4E).

It's interesting that FATS is a tumor suppressor highly expressed in testis, which is likely to be mediated by a transcriptional activator SRY (sex-determining region Y). SRY is a sex-determining gene on the mammalian Y chromosome and the founder member of the Sox (Sry-related HMG box) gene family [7,8,16]. Given that testis is an important organ for spermatogenesis and that a programmed DNA fragmentation and DNA damage response occur during the chromatin remodeling steps in spermatids [17,18], the high expression of FATS under transcriptional activation by p53 in spermatids may be critical to maintain genomic stability during spermiogenesis, which is extremely vulnerable to DNA damage. Our findings indicate that FATS is a novel target of p53 to achieve tighter control of p21 and stronger effect of cell-cycle arrest in surveillance of genomic stability.

## Abbreviations

FATS: fragile-site associated tumor suppressor; UTR: untranslated region; RE: response element; TSS: transcriptional start site; MEF: mouse embryonic fibroblast.

## Competing interests

The authors declare that they have no competing interests.

## Authors' contributions

XZ, QZ, JZ, LQ, SY, JF and ZL carried out the research. XH, YS and KHL participated in coordination and data analysis. ZL designed the study and drafted the manuscript. All authors read and approved the final manuscript.
